# Title Mania

**Published:** 1856-09

**Authors:** 


*Title Mania.*—The passion for titles among Americans has always been among the anomalies of our national character, and pointed at as one of the inconsistencies between the practice of the citizen, and the professions of our republicanism, which, in the rigid simplicity of its organization, ignores titles of all kinds, and professes to measure the standard of a man, by another rule than the varied shade of color of a ribbon, or the jewelry of a star. Practice, however, belies profession so much, that every other man in the community rejoices in some form of a title, and manages to wear his honors in all their glory to the grave. Honorables, Esquires, Generals, Captains, and the whole multitude of military titles are scattered throughout the community as thick as autumn leaves.

The medical profession has worn fewer *stars and stripes* probably than any other class of the community. They have been compelled to rest content with the appellation of doctor, though it may well be questioned how much of glory there shines in these degenerate days in the cognomen. Very few of the profession have probably sought other honors, for no greater honors, or victories have been achieved in any ranks, than those won in the daily warfares waged in life's battles by the physician.

But it has been left to these latter days for the aspirants for favor, and greater glories than are to be gathered in the quiet walks of every day life, to mark out a system of heraldic emblazonry for their adornment, and with which they cover themselves as with a garment.

We refer to the practice so prevalent at the present day among those who assume the dignities of medical authorship of loading down the title-pages of their works with a catalogue of the multitudinous honors which they sport. The name of every association or society to which they have ever belonged, the title to every book or paper they have written, is given with all the careful detail of an index page, as if they would astound the reader at first sight, with "behold, how great a man am I! question not my qualifications to write a book!"

Scarce a volume of late years has emanated from any of our eastern cities (and you *know all knowledge belongs* to the *east*, the *savans* all live *there!*) but has had affixed to the author's name a complete list of every labor and membership past, present and prospective of the candidates for renewed literary and scientific honors.

A good example of these kite-tail appendages has just fallen under our notice in a volume lately issued from a New York house. To judge from the display made, the erudition of the author must be truly astounding. Here it is:

"The —— ——. By —— —— ——, A. M., M. D., Permanent Member of the National Medical Association; Fellow of the New York Academy of Medicine; Member of the Massachusetts Medical Society; Member of the New York Pathological Society; Late Instructor on Diseases of Women and Children in the New York Preparatory School of Medicine; Physician for

Diseases of Women in the New York Northern Dispensary; Author of Monographs on Ergot; Uterine Hæmorrhage, Rupture of the Perinæum, &c., &c."

Particularly the &c., &c. What an amount of unrevealed glory is undoubtedly concealed beneath those two cabalistic substitutes for words, so generously repeated, too, to inform you that the end is not yet by any manner of means. How kindly considerate is the Doctor of his readers. A full revelation of all the honors won, would doubtless flash out such a flood of light, as to be dangerous to the vision, mental and corporal, and a veil is in kindness drawn across the scene, and you are permitted to look only as through a glass darkly.

Do you not think, Mr. Editor, if you or I, or any of our associates in the toils of medical bondage, dwelling at this milestone on the highway to the great west, should take it into our heads to give our knowledge an airing, and in order to give it an appearance of popularity should tack on to it the name of every local society we might happen to belong to, or the title of every squib we might have let off from time to time through the medical press, and leave much more to be guessed at than expressed, that our medical brethren dwelling where the morning sun first reveals himself, and where his light is supposed to be so much brighter and purer than out here where we breathe naught but fogs and miasms, would suffer a curvilinear elevation of the extremity of the nasal ornament to their countenances? I trow they would, and write it down as an evidence of western verdancy.

There are undoubtedly honors to which the medical man may attain, and which he may wear with a just pride. It should be the unceasing effort of every one to advance himself and his profession, and to the reward justly his due as a recompense for labors really performed, and advancements really made, he is more assuredly entitled. And there are uses of a reputation so gained that he may legitimately employ for his present and future benefit. But is it not in bad taste at least, to make such an ostentatious parade in public of every step that has been taken on the road to honor? What care the public for the name of every organization you have been, or may be connected with? Is it any positive evidence of ability that a physician may be, or is, a member of the medical societies of this city, or even that he represents a society in the American Medical Association, and obtains in the same a permanent membership? These great displays are certainly not devoid of the appearances of egotism, and I must confess that they always impress me with the idea that they are used simply to make the books sell.

TROCAR.

